# Evaluation of saliva and serum heme oxygenase, arylesterase and nuclear factor erythroid 2-related factor 2 levels in patients with stage III periodontitis: a cross sectional study

**DOI:** 10.1007/s00784-026-06814-x

**Published:** 2026-03-13

**Authors:** Zeynep Hazan Yildiz, Gülbahar Ustaoğlu, Emre Avci

**Affiliations:** 1 Department of Periodontology, University Of Health Sciences, Faculty of Gülhane Dentistry, Ankara, Turkey; 2 Department of Biochemistry, University Of Health Sciences, Faculty of Gülhane Pharmacy, Ankara, Turkey

**Keywords:** ARE, HO-1, NRF-2, Stage III periodontitis, Saliva, Serum

## Abstract

**Objectives:**

This study aimed to evaluate total oxidant status (TOS), total antioxidant status (TAS), oxidative stress index (OSI), arylesterase (ARE), heme oxygenase-1 (HO-1), and nuclear factor erythroid 2–related factor 2 (NRF-2) levels in saliva and serum samples of individuals with Stage III Grade B periodontitis, and to assess their relationship with disease activity and diagnostic potential in the pathogenesis of periodontitis.

**Materials and methods:**

Thirty-seven periodontally healthy individuals and thirty-seven patients with Stage III Grade B periodontitis were included in the study. After clinical measurements and sample collection ELISA method was used for analyses of TOS, TAS, OSI, ARE, HO-1, NRF-2 levels.

**Results:**

Salivary TAS, serum TAS, serum ARE, serum NRF-2, salivary HO-1 levels were significantly lower in periodontitis patients compared to the healthy control group (*p* = 0.015, p = < 0.001, *p* = 0.031, *p* = 0.041, *p* = 0.001). No significant difference was found in salivary and serum TOS, salivary and serum OSI, salivary ARE, salivary NRF-2, serum HO-1 levels (*p* = 0.685, *p* = 0.256, *p* = 0.146, *p* = 0.738, *p* = 0.513, *p* = 0.910, *p* = 0.256).

**Conclusion:**

Within the limitations of this study, the results suggest that decreased antioxidant capacity, particularly involving HO-1 and NRF-2, may contribute to oxidative stress–related tissue damage in periodontitis.

**Clinical relevance:**

Understanding the roles of HO-1 and NRF-2 in the antioxidant defense system provides novel insights into the biological mechanisms underlying periodontal tissue destruction. These biomarkers may help clinicians identify individuals with heightened oxidative stress and increased susceptibility to disease progression, enabling earlier diagnosis and more personalized, targeted therapeutic interventions to improve periodontal health outcomes.

## Introduction

Periodontitis is a chronic and progressive inflammatory disease characterized by the destruction of the tooth-supporting hard and soft tissues [1]. Periodontal diseases result from complex interactions between pathogenic bacteria and the host immuno-inflammatory responses. The subgingival biofilm, the primary etiologic factor in periodontal disease, stimulates neutrophils to produce reactive oxygen species (ROS) through a metabolic process known as respiratory burst. This process is catalyzed by NADPH oxidase during phagocytosis [2]. Excessive ROS generation or impaired antioxidant activity during periodontal disease cause oxidative damage to gingival tissues, the periodontal ligament, and alveolar bone. This damage occurs via multiple mechanisms, such as DNA damage, lipid peroxidation (LPO), protein oxidation, enzyme degradation, and induction of pro-inflammatory cytokine release, ultimately leading to tissue destruction and osteoclastic bone resorption [3–6]. Disruption of the oxidant–antioxidant balance, characterized by decreased antioxidant capacity and increased oxidative markers, has been identified as a key factor contributing to oxidative stress–mediated tissue damage and the pathogenesis of periodontitis [7, 8].

Oxidative stress can be effectively assessed through total oxidant status (TOS) and total antioxidant status (TAS). These parameters provide practical and comprehensive evaluations by considering both the additive effects of oxidants and the interactive nature of antioxidants. These approaches offer more practical and affordable alternatives to measuring multiple oxidant and antioxidant components separately, and the oxidative stress index (OSI), calculated from the TOS/TAS ratio, provides a better characterization of the oxidant–antioxidant imbalance [9]. Studies have shown that oxidative stress, caused by decreased antioxidant levels and increased oxidative markers, plays a role in the development of periodontal disease [10–13].

Among various oxidative stress–related enzymes, paraoxonase (PON) hydrolyzes phenylacetate and exhibits arylesterase (ARE) activity, which is highly sensitive to oxidative stress; oxidants can reduce ARE activity, highlighting its potential role as a biomarker of oxidative damage [14]. Heme oxygenase-1 (HO-1) plays an adaptive and defensive role in protecting cells against oxidative stress to maintain homeostasis. Factors such as hypoxia, hyperthermia, and radiation are known to activate HO-1, making it an important indicator of oxidative stress [15, 16]. Nuclear factor erythroid 2–related factor 2 (NRF-2) is a transcription factor that regulates HO-1 and orchestrates cellular defenses against oxidative stress–induced cytotoxicity [17]. Mechanistically, NRF-2 activation enhances antioxidant capacity by upregulating HO-1 and other protective genes, whereas HO-1 directly reduces oxidative damage and modulates inflammatory responses in periodontal tissues. Together, these molecules constitute a coordinated antioxidant defense system that may play a crucial role in limiting ROS-mediated tissue destruction in periodontitis [18, 19].

Despite increasing evidence linking oxidative imbalance to periodontitis, to the best of our knowledge, no previous study has investigated the role of HO-1 and NRF-2 in the pathogenesis of periodontitis. Therefore, this study aimed to evaluate the levels of TAS, TOS, OSI, ARE, HO-1, and NRF-2 in saliva and serum samples from periodontally healthy individuals and patients with Stage III Grade B periodontitis to determine their relationship with disease activity and potential diagnostic value.

Taken together, these findings suggest that oxidative imbalance and antioxidant defense mechanisms, particularly those involving HO-1 and NRF-2, may play key roles in periodontal tissue destruction. Therefore, we hypothesize that alterations in salivary and serum HO-1 and NRF-2 levels are associated with disease activity in Stage III Grade B periodontitis and may serve as potential biomarkers for disease progression.

## Materials and methods

### Participants

This study was ethically approved by the Health Sciences University Clinical Research Ethics Committee (decision number 2023/82) and registered at ClinicalTrials.gov (NCT06986044).

A priori power analysis was performed using G*Power software (version 3.1). Based on previous studies investigating oxidative stress biomarkers in periodontitis, it was determined that at least 36 participants per group were required to achieve a power of 95% with α = 0.05. Therefore, 37 subjects were included in each group. The effect size calculated for serum TAS levels (Cohen’s d = 0.83) indicated a large effect, confirming that the sample size was sufficient [10].

All patients who were invited to participate in the study provided written informed consent. The study was conducted at the Department of Periodontology, Faculty of Dentistry, University of Health Sciences, between May 2023 and May 2024. Study groups were classified according to the 2017 World Workshop’s new classification system for Periodontal and Peri-implant Diseases and Conditions, as outlined in the consensus report [20]. A total of 74 participants were enrolled, comprising 37 periodontally healthy individuals and 37 patients diagnosed with Stage III, Grade B periodontitis.

Participants were excluded if they had any systemic condition that could influence periodontal status, including cardiovascular disease, autoimmune disorders, rheumatoid arthritis, diabetes mellitus, or a history of chemotherapy or radiotherapy. Additional exclusion criteria comprised the use of medications known to affect periodontal tissues, fewer than 20 remaining natural teeth (excluding third molars), loss of five or more teeth due to periodontal disease, a history of periodontal therapy, or use of antibiotics, anti-inflammatory, or immunosuppressive agents within the past six months. Individuals who were smokers, under 18 or over 65 years of age, pregnant, or lactating at the time of examination were also excluded.

### Clinical examination

Clinical periodontal variables were recorded by a single periodontist (Z.H.Y.). Periodontal measurements, including plaque index (PI) [21], gingival index (GI) [22], probing depth (PD), clinical attachment level (CAL), and bleeding on probing (BoP), were performed using a Williams-type periodontal probe and all measurements were recorded at four sites per tooth (mesial, distal, buccal, and lingual).

The criteria for individuals who were periodontally healthy were the mean of the percentage of BoP 10%, but with no sites of attachment loss. The criteria for stage III grade B periodontitis included patients having at PD ≤ 6 mm, CAL ≥ 5 mm, tooth loss related to periodontitis ≤ 4, radiographic bone loss to age ratio (RBL/AGE) between 0.25 and 1 and periodontal tissue destruction compatible with biofilm accumulation.

### Saliva collection

For the collection of unstimulated saliva samples, a clinical environment in which patients felt comfortable was selected. Participants were instructed to refrain from eating, drinking, or using any oral hygiene products for at least 2 h prior to sample collection. To minimize circadian rhythm-related variability, all samples were collected between 9:00 and 11:00 a.m. Patients were asked to rinse their mouths with water, accumulate saliva in the floor of the mouth for 5 min, and then gently transfer it into a sterile plastic collection tube. The collected unstimulated saliva samples were centrifuged at 3.000 rpm for 10 min and subsequently transferred to 1.5 mL Eppendorf tubes and stored at − 80 °C until the day of analysis [23].

### Serum collection

Standard venipuncture was performed for venous blood sample were collected from the antecubital fossa with the patients in a sitting position. The samples were centrifuged at 4000 rpm for 3 min, serum samples were separated and transferred into 1.5 ml Eppendorf tubes and stored at −80 °C until the day of analysis.

### Biochemical methodology

TAS concentrations were evaluated utilizing an innovative automated colorimetric assay, originally developed by Erel [24]. The outcomes were reported as millimoles of Trolox equivalent per liter (mmol Trolox Eq/L).

TOS concentrations were determined through a distinct automated colorimetric technique, also described by Erel [9]. Results were expressed as micromoles of hydrogen peroxide equivalent per liter (µmol H₂O₂ Eq/L).

OSI was calculated to represent the balance between oxidative and antioxidative markers [25]. For this purpose, TAS values (initially in mmol Trolox Eq/L) were converted to µmol Trolox Eq/L. OSI was then computed using the following formula: OSI = [(TOS, µmol/L)/(TAS, µmol Trolox Eq/L)] × 100.

Arylesterase activity was assessed using commercially available assay kits (Rel Assay Diagnostics, Turkey). For the measurement of arylesterase activity, phenylacetate was used as the substrate. The enzymatic activity was calculated based on the molar extinction coefficient of the phenol formed (1,310 M⁻¹cm⁻¹). One unit of arylesterase activity was defined as the amount of enzyme required to hydrolyze 1 µmol of phenol per minute under the assay conditions and was also expressed as U/L.

The level of NRF-2 was quantified using an enzyme-linked immunosorbent assay (ELISA) kit (BT lab.). In this assay, wells were pre-coated with human NRF-2-specific antibodies. Samples containing NRF2 were added to the wells, allowing target binding. Subsequently, a biotin-conjugated anti-NRF-2 antibody was applied, followed by streptavidin-horseradish peroxidase (HRP) conjugate. After washing to remove unbound components, a substrate solution was added, producing a colorimetric reaction proportional to the amount of NRF-2 present. The reaction was terminated by adding an acidic stop solution, and absorbance was measured at 450 nm.

The concentration of HO-1 was measured using an ELISA kit (BT Lab.) Wells were pre-coated with human HO-1-specific antibodies. Following the addition of samples, human HO-1 proteins bound to the immobilized antibodies. A biotinylated anti-HO-1 antibody and subsequently streptavidin-HRP conjugate were introduced. After removal of unbound components via washing, a substrate solution was added to initiate color development, which was proportional to the HO-1 concentration. The reaction was stopped using an acidic solution, and absorbance was recorded at 450 nm.

NRF2 and HO-1 levels were determined using commercially available human ELISA kits from BT-Lab (Wuhan, China; Cat. No: E3244Hu for NRF2 and E0932Hu for HO-1). According to the manufacturer’s datasheets, the sensitivity of the NRF2 kit is 0.11 ng/mL with an assay range of 0.2–60 ng/mL, while the HO-1 kit shows a sensitivity of 0.05 ng/mL and an assay range of 0.1–20 ng/mL. Both assays are based on a sandwich ELISA principle. The intra-assay and inter-assay coefficients of variation (CV) reported by the manufacturer are **<** 8% and < 10%, respectively. All measurements were performed using a Bio-Tek EL X 800 microplate reader and an EL X 50 automatic strip washer, following the manufacturer’s protocols.

### Statistical analysis

All statistical analyses were performed using SPSS (version 27.0; IBM, Chicago, IL, USA). Data distribution was evaluated with the Kolmogorov–Smirnov test. For normally distributed variables, group comparisons were conducted using the Independent Samples t-test, while the Mann–Whitney U test was used for non-normally distributed variables. Effect sizes (Cohen’s d or rank-biserial correlation) and 95% confidence intervals were calculated. Correlations between biochemical and clinical parameters were assessed using Spearman’s rank correlation, given that several variables did not satisfy parametric assumptions. To account for multiple comparisons across biomarkers, Bonferroni correction was applied, yielding an adjusted α level of 0.004. A p-value < 0.05 was considered statistically significant before correction.

## Results

### Clinical and demografik data

In this study, 37 periodontally healthy individuals (19 females, 18 males) and 37 individuals diagnosed with Stage III, Grade B periodontitis (20 females, 17 males) were included. The mean age was 39.48 ± 9.78 years in the periodontally healthy group and 39.05 ± 10.17 years in the periodontitis group. The demographic characteristics of the participants are present in Table 1. There were no statistically significant differences between the groups in terms of age or gender (*p* > 0.05).

**Table 1:** Demographic Characteristics of the Study Participants

	Healthy (*n* = 37)	Periodontitis (*n* = 37)	*p*-value
Age mean ± SD	39.48±9.78	39.05±10.17	0.853
Gender Female n(%) Male n(%)	19(51.35) 18(48.65)	20(54.05) 17(45.95)	0.816

Mean ± SD, mean ± standart derivation

Periodontal parameters for the study groups were assessed based on full-mouth examinations. The median (minimum–maximum) values of the PI, GI, PD, CAL, and BoP are present in Table 2. All clinical parameters were significantly higher in the periodontitis group compared to the periodontally healthy group (*p* < 0.01).

**Table 2:** Comparison of Clinical Periodontal Parameters Between Groups

	Healthy (*n* = 37)	Periodontitis (*n* = 37)	*p*-value
PI (min-max) median	0 0	(0.9–2.3) 1.7	**<0.001**
GI (min-max) median	0 0	(1.2–4.5) 1.4	**<0.001**
PD (mm) (min-max) median	(1.4–2.0.4.0) 1.7	(2.0–5.1.0.1) 2.8	**<0.001**
CAL (mm) (min-max) median	(1.4–2.0.4.0) 1.7	(2.1–5.2) 2.8	**<0.001**
BoP (%) (min-max) median	0 0	(28.7–86.4) 43.8	**<0.001**

Statistically significant differences between the groups are indicated in bold (*p* < 0.05)

*PI* plaque index, *GI* gingival index, *PD* probing depth, *CAL* clinical attachment level, *BoP* bleeding on probing

### Laboratory data

**Fig. 1:**
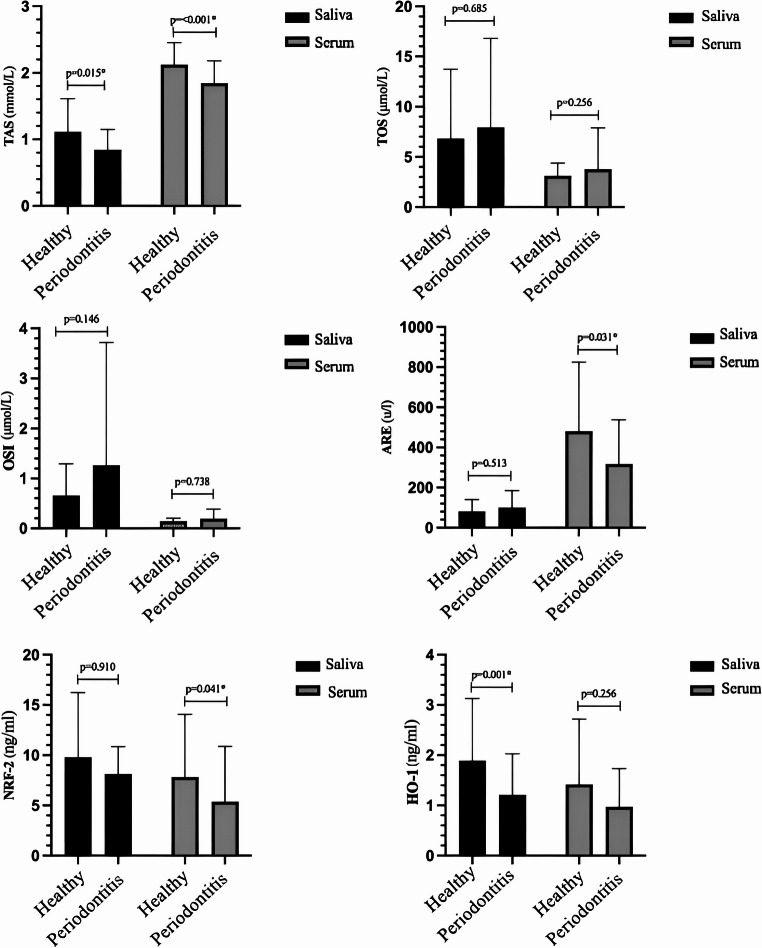
Comparison of Salivary and Serum Biomarker Levels Between Periodontitis and Healthy Groups

Serum and salivary TAS levels were significantly higher in the periodontally healthy group compared to the periodontitis group (*p* < 0.001 and *p* = 0.015, respectively). Serum ARE levels were also significantly higher in the periodontally healthy group (*p* = 0.031). No significant differences were observed between the groups in terms of salivary ARE, salivary and serum TOS, or OSI levels (*p* = 0.513, *p* = 0.685, *p* = 0.256, *p* = 0.146, and *p* = 0.738, respectively). Serum NRF-2 and salivary HO-1 levels were significantly higher in the periodontally healthy group than in the periodontitis group (*p* = 0.041 and *p* = 0.001, respectively). However, no significant differences were found in salivary NRF-2 or serum HO-1 levels between the groups (*p* = 0.910 and *p* = 0.256, respectively). Mean differences between the groups are illustrated in Fig. 1, and the median values along with their ranges (min–max) are summarized in Table 3.

**Table 3:** Comparison of Salivary and Serum Biomarkers Between the Periodontitis and Healthy Groups

	Healthy (*n* = 37) median (min-max)	Periodontitis (*n* = 37) median (min-max)	*p*-value
Saliva TAS (µmol/L)	0.97 (0.27–2.44)	0.82 (0.14–1.57)	**0.015**
Serum TAS (µmol/L)	2.17 (0.76–2.68)	1.86 (0.85–2.71)	**< 0.001**
Saliva TOS (µmol/L)	5.22 (0.30–32.85.30.85)	4.87 (0.94–46.83)	0.685
Serum TOS (µmol/L)	3.22 (0.28–19.63)	2.42 (0.98–19.63)	0.256
Saliva OSI	0.40 (0.03–3.17)	0.55 (0.13–14.48)	0.146
Serum OSI	0.15 (0.02–0.30)	0.13 (0.05–1.10)	0.738
Saliva ARE (U/L)	77.10 (6.10–257.60.10.60)	77.20 (14.10–378.20.10.20)	0.513
Serum ARE (U/L)	479.70 (90.30–1376.0)	191.60 (85.80–852.60.80.60)	**0.031**
Saliva NRF-2 (ng/ml)	7.79 (2.13–29.17)	7.82 (2.61–15.85)	0.910
Serum NRF-2 (ng/ml)	5.03 (1.21–27.93)	3.01 (1.65–21.07)	**0.041**
Saliva HO-1 (ng/ml)	1.54 (0.28–6.29)	0.87 (0.36–4.51)	**0.001**
Serum HO-1 (ng/ml)	0.89 (0.18–5.09)	0.62 (0.24–3.37)	0.256

Statistically significant differences between the groups are indicated in bold (*p* < 0.05)

*TAS* total antioxidant status, *TOS* total oxidant status, *OSI* oxidative stress index, *ARE* arylesterase, *NRF-2* nuclear factor erythroid 2 related factor 2, *HO-1* heme oxygenase-1

### Correlations

The correlations between clinical parameters and biomarkers in all participants were analyzed using Spearman’s rank correlation test and are summarized in Table 4. A weak negative correlation was observed between the PI and salivary TAS levels, while strong negative correlations were found between PI and both serum TAS and salivary HO-1 levels. The GI showed strong negative correlations with serum TAS, serum ARE, and salivary HO-1 levels. PD and CAL demonstrated strong negative correlations with serum TAS and salivary HO-1 levels, and a weak negative correlation with serum NRF-2 levels. BoP was strongly negatively correlated with serum TAS and salivary HO-1, and showed a weak negative correlation with serum ARE levels.

**Table 4:** Spearman Correlations Between Clinical Parameters and Biomarkers in All Participants

	PI	GI	PD	CAL	BoP
TAS Saliva (mmol/L)	−0.278*	−0.225	−0.142	−0.146	−0.228
TAS Serum (mmol/L)	−0.413**	−0.404**	−0.366**	−0.351**	−0.382**
TOS Saliva (µmol/L)	0.033	0.069	0.051	0.043	0.044
TOS Serum (µmol/L)	−0.118	−0.097	−0.157	−0.144	−0.076
OSI Saliva	0.152	0.164	0.112	0.106	0.141
OSI Serum	0.035	0.039	−0.016	−0.007	0.050
ARE Saliva (u/L)	0.177	0.016	0.055	0.062	0.066
ARE Serum (u/L)	−0.206	−0.320**	−0.225	−0.217	−0.262*
NRF-2 Saliva (ng/ml)	0.02	−0.006	−0.091	−0.085	−0.019
NRF-2 Serum (ng/ml)	−0.155	0.194	−0.261**	0.254*	−0.192
HO-1 Saliva (ng/ml)	−0.384**	−0.380**	−0.384**	−0.379**	−0.380**
HO-1 Serum (ng/ml)	−0.088	−0.073	−0.190	−0.190	−0.083

Statistically significant differences are indicated in bold (p<0.05) *: weak correlation **: strong correlation

Correlation analysis of biomarkers for all participants were also performed using Spearman’s rank correlation test and is presented in Table 5. In the whole group, a weak positive correlation was observed between salivary TAS levels and both salivary TOS levels and serum NRF-2 levels, while a strong positive correlation was found with serum HO-1 levels. Salivary TOS levels showed a strong positive correlation with salivary OSI levels and a weak negative correlation with serum ARE levels. A strong positive correlation was also observed between salivary and serum ARE levels. Additionally, salivary NRF-2 levels were strongly positively correlated with salivary HO-1 levels, and salivary HO-1 levels showed a weak positive correlation with serum TAS levels. Serum TAS levels had a strong positive correlation with serum TOS levels, and serum TOS levels were strongly positively correlated with serum OSI levels. Finally, serum NRF-2 levels showed a strong positive correlation with serum HO-1 levels.

**Table 5:** Spearman Correlations Among Salivary And Serum Biomarkers in All Participants

	TAS Saliva (mmol/L)	TAS Serum (mmol/L)	TOS Saliva (µmol/L)	TOS Serum (µmol/L)	OSI Saliva	OSI Serum	ARE Saliva (u/L)	ARE Serum (u/L)	NRF-2 Saliva (ng/ml)	NRF-2 Serum (ng/ml)	HO-1 Saliva (ng/ml)	HO-1 Serum (ng/ml)
TAS Saliva (mmol/L)		0.209	0.252*	0.004	−0.098	−0.062	0.008	−0.15	−0.195	0.234*	−0.168	0298**
TAS Serum (mmol/L)	0.209		0.083	0.413**	0.013	0.064	−0.09	0.179	0.072	0.216	0.275*	0.096
TOS Saliva (µmol/L)	0.252*	0.083		−0.155	0.920**	−0. 188	−0.172	−0.246*	0.083	0.065	0.025	0.198
TOS Serum (µmol/L)	0.004	0.413**	−0.155		−0.139	0.900**	0.122	0.228	0.100	0.117	0.087	0.072
OSI Saliva	−0.098	0.013	0.920**	−0.139		−0. 142	−0.164	−0. 182	0.145	−0.010	0.063	0.091
OSI Serum	−0.062	0.064	−0.188	0.900**	−0.142		0.157	0.201	0.06	0.061	−0.033	0.056
ARE Saliva (u/L)	0.008	−0.090	−0.172	0.122	−0.164	0.157		0.466**	0.017	0.063	0.015	−0.058
ARE Serum (u/L)	−0.15	0.179	−0.246*	0.228	−0.182	0.201	0.466**		0.038	−0.084	0.205	−0.144
NRF-2 Saliva (ng/ml)	−0.195	0.072	0.083	0.100	0.145	0.06	0.017	0.038		0.130	0.559**	0.005
NRF-2 Serum (ng/ml)	0.234*	0.216	0.065	0.117	−0.01	0.061	0.063	−0.084	0.130		0.101	0.777**
HO-1 Saliva (ng/ml)	−0.168	0.275*	0.025	0.087	0.063	−0.033	0.015	0.205	0.559**	0.101		−0. 148
HO-1 Serum (ng/ml)	0.298*	0.096	0.198	0.072	0.091	0.056	−0.058	−0.144	0.005	0.777**	−0.148	

Statistically significant differences are indicated in bold (*p*<0.05) *: weak correlation **: strong correlation

*TAS* total antioxidant status, *TOS* total oxidant status, *OSI* oxidative stress index, *ARE* arylesterase, *NRF-2* nuclear factor erythroid 2 related factor 2, *HO-1* heme oxygenase-1

## Discussion

According to the literature, oxidative stress is a major contributor to the pathogenesis of periodontal diseases, and periodontal treatment can help normalize relevant biomarkers and potentially return them to levels observed in periodontally healthy individuals [25–27].

Measuring each oxidant and antioxidant molecule separately is often impractical and time-consuming, and may not accurately reflect the overall redox balance in biological systems [28, 29]. Therefore, assessing TOS offers a more practical and integrated approach to estimate overall oxidative stress. In our study, no statistically significant difference was observed in serum or salivary TOS levels between the periodontitis and healthy groups. These findings are consistent with several recent cross-sectional studies that also reported comparable TOS levels between periodontitis and control groups [30, 31]. This lack of difference may be attributed to the complex biochemical composition of saliva, variations in flow rate, and external factors such as dietary habits, medication use, and oral hygiene, all of which can influence TOS measurements [13, 32]. Although the slightly elevated TOS levels observed in our study may suggest a systemic oxidative burden in periodontitis, previous research indicates that oxidative stress increases both locally and systemically in periodontal disease [12]. Taken together, these findings suggest that oxidative stress, as reflected by TOS levels, may exert both local and systemic effects, contributing to the multifactorial pathogenesis of periodontal disease.

TAS reflects the overall antioxidant defense against ROS [33].]. In our study, statistically significant higher serum TAS levels were observed in the healthy group. Consistent with our results, Chapple et al. reported that TAS levels reflect alterations in the antioxidant system during the pathogenesis of periodontal disease and demonstrated decreased TAS levels in periodontitis patients [34]. Similarly, Atagün et al. [35] found reduced TAS levels in both the serum and saliva of periodontitis patients, while Baltacıoğlu et al. [36] reported markedly lower TAS levels in both serum and gingival crevicular fluid (GCF). However, in contrast to these findings, some studies have reported higher TAS levels in periodontitis patients compared to healthy individuals [37, 38]. This has been interpreted as a compensatory antioxidant response to increased oxidative stress, suggesting that high oxidative damage may stimulate antioxidant production [39]. TAS levels may transiently increase during the initial stages of oxidative stress as a compensatory mechanism, but tend to decline with sustained ROS production. These inconsistencies may reflect the complex interplay among antioxidant systems, sample types, methodological variability, and patient-related factors such as diet or systemic conditions [40, 41].

OSI, calculated as the TOS/TAS ratio, is regarded as a more comprehensive and reliable indicator of overall oxidative stress [9]. In our study, OSI values did not differ significantly between the healthy and periodontitis groups, suggesting that the compensatory antioxidant response may have counterbalanced the oxidative burden at a systemic level. However, our findings supported the association between periodontal disease and imbalance in redox homeostasis, consistent with previous studies [42–44].

ARE, an enzyme involved in the detoxification of lipid and glucose oxidation products, plays a crucial role in antioxidant defense system [45]. In the present study, serum ARE levels were significantly higher in healthy individuals compared to those with periodontitis, suggesting a reduction in enzymatic antioxidant activity in the disease group. Similarly, in a cross-sectional study involving patients with psoriasis, Paksoy et al. [11] reported higher salivary ARE levels in the healthy group compared to the periodontitis group, which is consistent with the findings of our study. The significant difference in serum ARE levels observed in our study suggests that ARE may exert a more pronounced systemic antioxidant effect compared to its local role.

HO enzymes, key components of the antioxidant defense system, play a critical role in heme catabolism, and particularly HO-1 is induced as an adaptive stress-response enzyme [46, 47]. HO-1 protects tissue damage by mechanisms such as antioxidation, anti-inflammation and anti-apoptosis and has been shown to have a protective role against oxidative stress [48]. To the best of our knowledge, this is the first study to evaluate HO-1 levels in both saliva and serum samples of patients with periodontitis. Previous research has demonstrated that HO-1 contributes to periodontal tissue protection by modulating inflammatory and immune responses. HO-1 has been reported to downregulate RANKL expression in periodontal ligament cells [49], show elevated expression in the gingival tissues of smokers with periodontitis [50], and suppress inflammatory responses, including TNF-α and LPS-induced cytokine production [51–54]. Collectively, these findings suggest that HO-1 activation may serve as an endogenous defense mechanism that mitigates inflammation and tissue injury, highlighting its potential as a therapeutic target in periodontal disease. In our study, both serum and salivary HO-1 levels were higher in the healthy control group, with salivary levels showing a statistically significant difference. A strong negative correlation was observed between salivary HO-1 levels and clinical periodontal parameters. The marked elevation in salivary HO-1 levels may reflect a rapid local defense response and enhanced tissue activity within oral biochemical and immunological processes. In our study, both serum and salivary HO-1 levels were higher in the healthy control group, with salivary levels showing a statistically significant difference. A strong negative correlation was observed between salivary HO-1 levels and clinical periodontal parameters. The marked increase in salivary HO-1 levels may reflect a rapid local defense response and heightened tissue activity within oral biochemical and immunological processes.

NRF-2 is a key transcription factor that regulates antioxidant responses and plays a critical role in neutrophil function, particularly in individuals with periodontitis [55–57]. Elucidating the regulatory role of NRF-2 in immune cell function and cytokine expression during the pathogenesis of periodontitis could provide valuable insights for developing novel antioxidant and anti-inflammatory therapeutic strategies [58]. In healthy individuals, the regulation of oxidative stress within periodontal tissues suggests that NRF-2 is essential for protecting these tissues against the constant challenge of bacterial pathogens and immune cell activity [59]. To the best of our knowledge, this is the first study to evaluate NRF-2 levels in both serum and saliva samples of patients with periodontitis.

In our study, serum NRF-2 levels were significantly higher in the healthy group, while salivary levels showed no significant difference. Interestingly, strong positive correlations were observed between salivary and serum levels of both NRF-2 and HO-1. Previous experimental studies demonstrated that bioactive compounds such as resveratrol and isorhamnetin enhance NRF-2-dependent HO-1 expression in gingival fibroblasts, thereby suppressing pro-inflammatory cytokine release and attenuating periodontal tissue destruction. Collectively, these findings underscore the pivotal role of the NRF-2/HO-1 signaling pathway in modulating oxidative stress and inflammation during the pathogenesis of periodontitis [18, 60, 61]. Based on our results, HO-1 appears to act as a more responsive local biomarker of oxidative stress and inflammation, whereas NRF-2 may exert a stronger systemic effect, possibly through its association with circulating polymorphonuclear leukocytes. Overall, these results further support the functional relevance of the NRF-2/HO-1 signaling axis in maintaining redox balance and protecting periodontal tissues from oxidative and inflammatory damage.

The observed reduction in serum NRF-2 levels in periodontitis patients may reflect impaired antioxidant transcriptional activity, possibly due to the chronic inflammatory burden and persistent neutrophil activation characteristic of the disease. In contrast, the elevated local HO-1 response highlights its critical role in regulating oxidative stress at the tissue level.

This study has several limitations that should be taken into account when interpreting the findings. First, the cross-sectional design limits the ability to establish causal inferences between antioxidant profiles and periodontal health. Second, although serum and saliva samples were selected for their non-invasive nature and feasibility for larger volume collection, they may not fully capture site-specific oxidative alterations occurring within periodontal tissues. Third, the relatively limited sample size and potential confounding factors such as age, gender, and dietary habits could have influenced the oxidative and inflammatory parameters. Future longitudinal and interventional studies, particularly those integrating GCF biomarkers and assessing treatment outcomes, are warranted to better elucidate the diagnostic and prognostic potential of oxidative stress–related markers in periodontitis.

## Conclusion

In conclusion, our findings reinforce the involvement of oxidative stress in the pathogenesis of periodontitis, characterized by both systemic and local disturbances in oxidant–antioxidant balance. HO-1 appears to serve as a sensitive local marker of oxidative stress, whereas NRF-2 may better reflect systemic antioxidant capacity, underscoring the complementary roles of these biomarkers in redox regulation. Further prospective and interventional studies are warranted to assess the responsiveness of these biomarkers to periodontal therapy and to clarify their diagnostic, prognostic, and therapeutic monitoring potential. Given the cross-sectional nature of the present study, these associations should be interpreted as correlational rather than causal. Collectively, these results highlight the potential clinical relevance of oxidative stress–related markers as adjunctive tools for monitoring periodontal disease activity and guiding personalized treatment strategies, within the limitations of our study.

## Data Availability

The data of the current study are available from the corresponding author on reasonable request registered at ClinicalTrials.gov (NCT06986044).
